# Supplementary figures and images for: Ultrastructure of light-activated axons following optogenetic stimulation to produce late-phase long-term potentiation
Source: PLoS One. 2020 Jan 15;15(1):e0226797. doi: 10.1371/journal.pone.0226797 (PMC6961864; doi:10.1371/journal.pone.0226797)

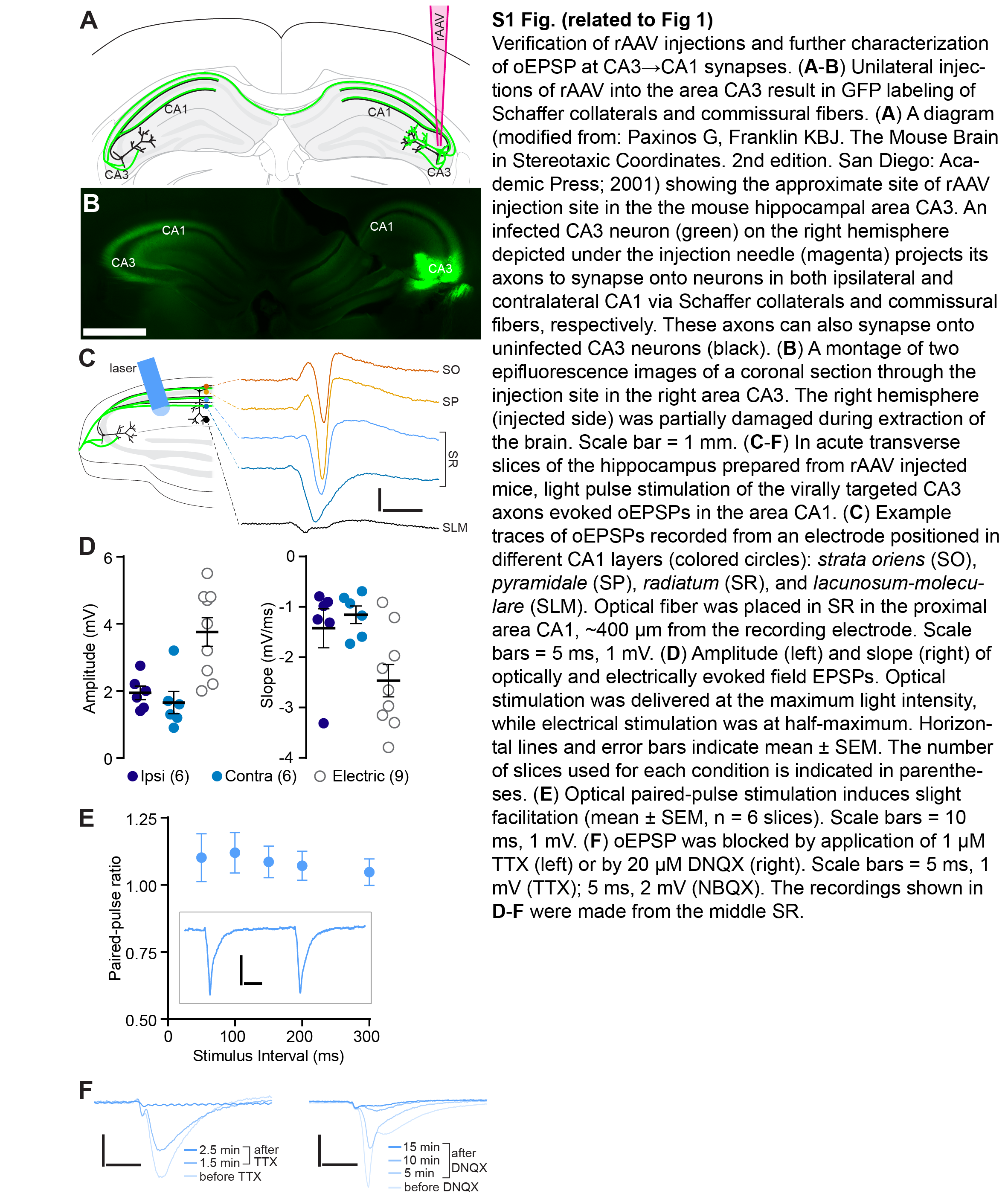

Supplement: S1 Fig — (A-B) Unilateral injections of rAAV into the area CA3 result in GFP labeling of Schaffer collaterals and commissural fibers. (A) A diagram (modified from: Paxinos G, Franklin KBJ. The Mouse Brain in Stereotaxic Coordinates. 2nd edition. San Diego: Academic Press; 2001) showing the approximate site of rAAV injection site in the mouse hippocampal area CA3. An infected CA3 neuron (green) on the right hemisphere depicted under the injection needle (magenta) projects its axons to synapse onto neurons in both ipsilateral and contralateral CA1 via Schaffer collaterals and commissural fibers, respectively. These axons can also synapse onto uninfected CA3 neurons (black). (B) A montage of two epifluorescence images of a coronal section through the injection site in the right area CA3. The right hemisphere (injected side) was partially damaged during extraction of the brain. Scale bar = 1 mm. (C-F) In acute transverse slices of the hippocampus prepared from rAAV injected mice, light pulse stimulation of the virally targeted CA3 axons evoked oEPSPs in the area CA1. (C) Example traces of oEPSPs recorded from an electrode positioned in different CA1 layers (colored circles): strata oriens (SO), pyramidale (SP), radiatum (SR), and lacunosum-moleculare (SLM). Optical fiber was placed in SR in the proximal area CA1, ~400 μm from the recording electrode. Scale bars = 5 ms, 1 mV. (D) Amplitude (left) and slope (right) of optically and electrically evoked field EPSPs. Optical stimulation was delivered at the maximum light intensity, while electrical stimulation was at half-maximum. Horizontal lines and error bars indicate mean ± SEM. The number of slices used for each condition is indicated in parentheses. (E) Optical paired-pulse stimulation induces slight facilitation (mean ± SEM, n = 6 slices). Scale bars = 10 ms, 1 mV. (F) oEPSP was blocked by application of 1 μM TTX (left) or by 20 μM DNQX (right). Scale bars = 5 ms, 1 mV (TTX); 5 ms, 2 mV (NBQX). The recordings shown in D-F were [file pone.0226797.s001.tif]

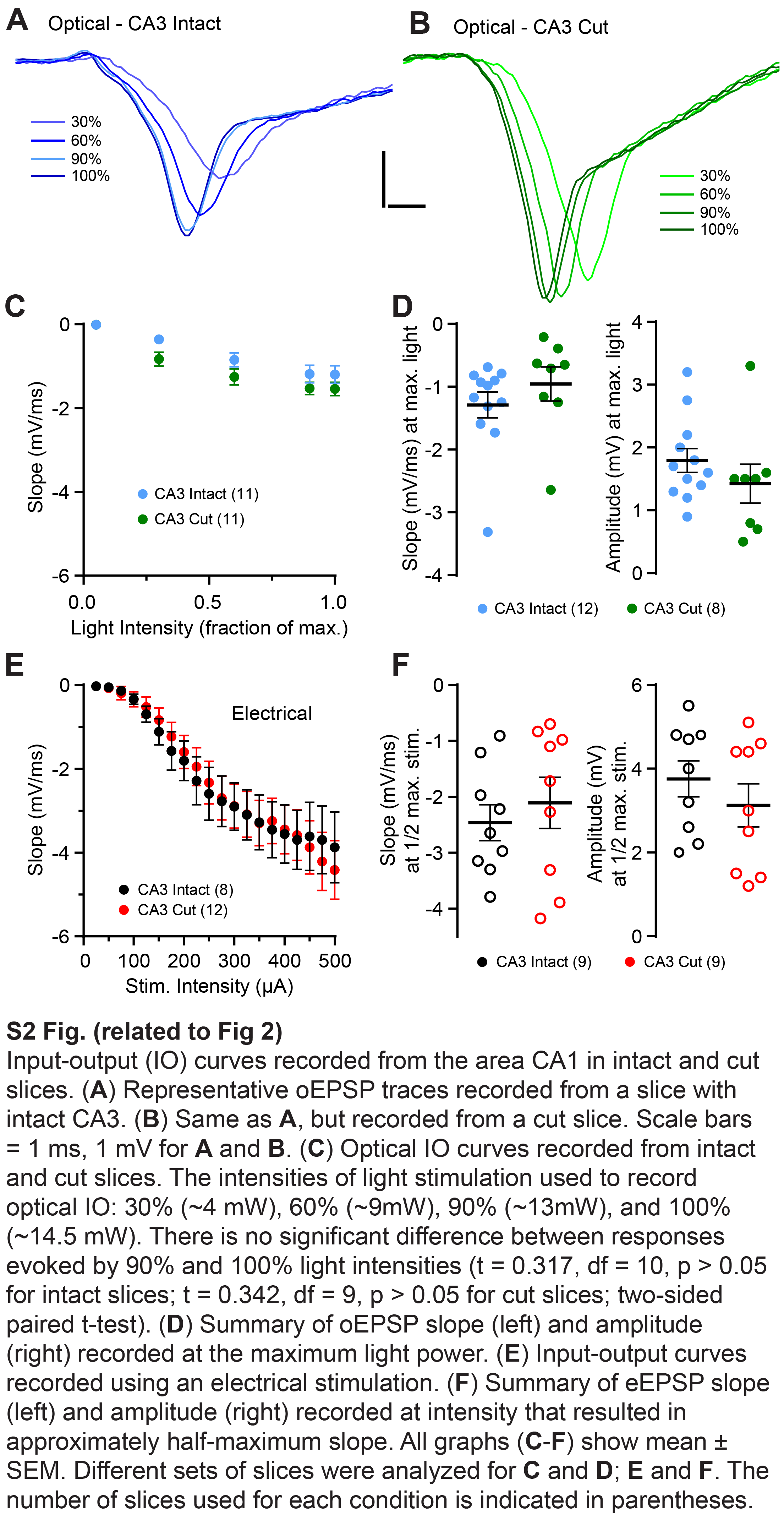

Supplement: S2 Fig — (A) Representative oEPSP traces recorded from a slice with intact CA3. (B) Same as A, but recorded from a cut slice. Scale bars = 1 ms, 1 mV for A and B. (C) Optical IO curves recorded from intact and cut slices. The intensities of light stimulation used to record optical IO: 30% (~4 mW), 60% (~9mW), 90% (~13mW), and 100% (~14.5 mW). There is no significant difference between responses evoked by 90% and 100% light intensities (t = 0.317, df = 10, p > 0.05 for intact slices; t = 0.342, df = 9, p > 0.05 for cut slices; two-sided paired t-test). (D) Summary of oEPSP slope (left) and amplitude (right) recorded at the maximum light power. (E) Input-output curves recorded using an electrical stimulation. (F) Summary of eEPSP slope (left) and amplitude (right) recorded at intensity that resulted in approximately half-maximum slope. All graphs (C-F) show mean ± SEM. Different sets of slices were analyzed for C and D; E and F. The number of slices used for each condition is indicated in parentheses. (TIF) [file pone.0226797.s002.tif]

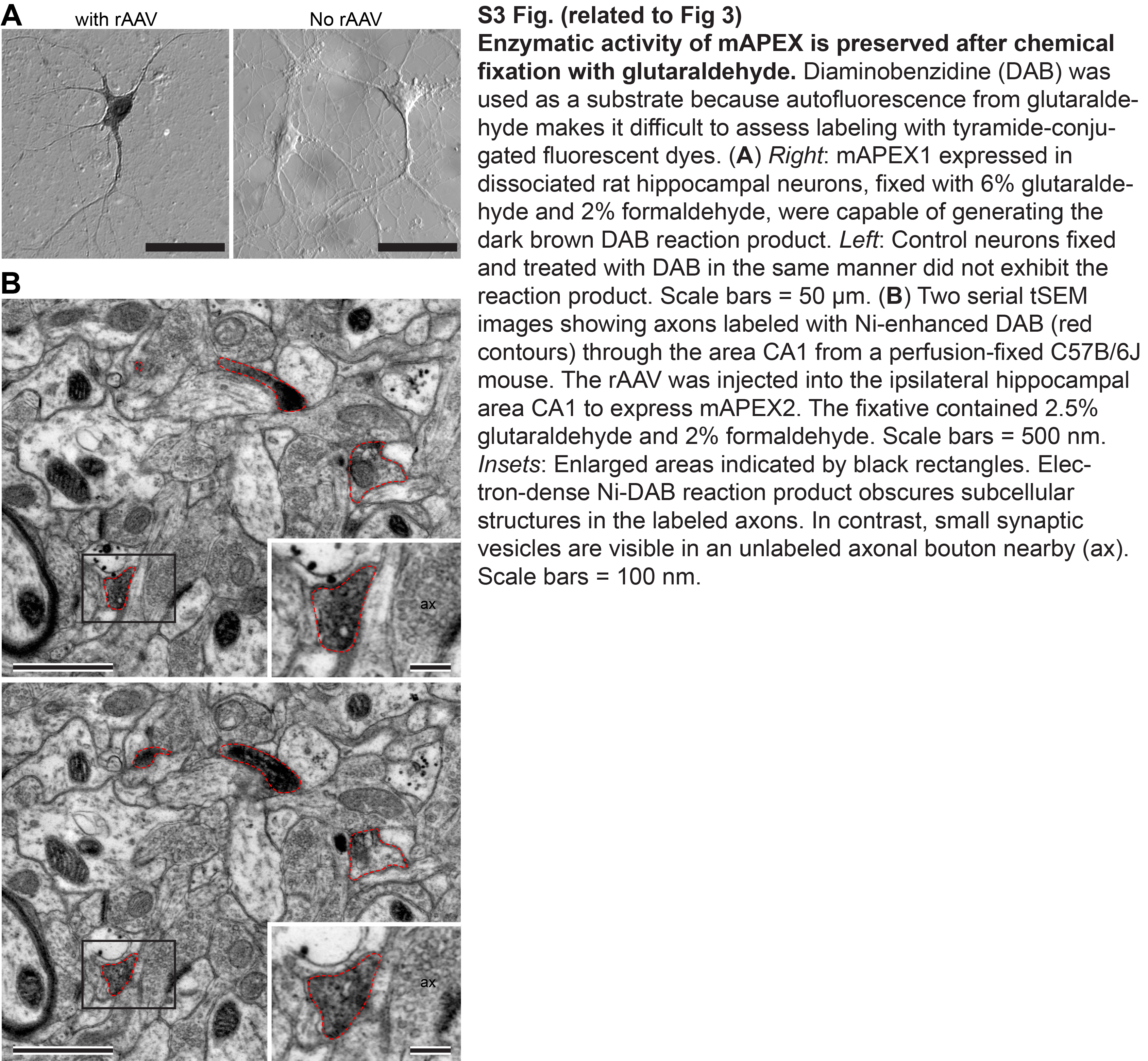

Supplement: S3 Fig — Diaminobenzidine (DAB) was used as a substrate because autofluorescence from glutaraldehyde makes it difficult to assess labeling with tyramide-conjugated fluorescent dyes. (A) Right: mAPEX1 expressed in dissociated rat hippocampal neurons, fixed with 6% glutaraldehyde and 2% formaldehyde, were capable of generating the dark brown DAB reaction product. Left: Control neurons fixed and treated with DAB in the same manner did not exhibit the reaction product. Scale bars = 50 μm. (B) Two serial tSEM images showing axons labeled with Ni-enhanced DAB (red contours) through the area CA1 from a perfusion-fixed C57B/6J mouse. The rAAV was injected into the ipsilateral hippocampal area CA1 to express mAPEX2. The fixative contained 2.5% glutaraldehyde and 2% formaldehyde. Scale bars = 500 nm. Insets: Enlarged areas indicated by black rectangles. Electron-dense Ni-DAB reaction product obscures subcellular structures in the labeled axons. In contrast, small synaptic vesicles are visible in an unlabeled axonal bouton nearby (ax). Scale bars = 100 nm. (TIF) [file pone.0226797.s003.tif]

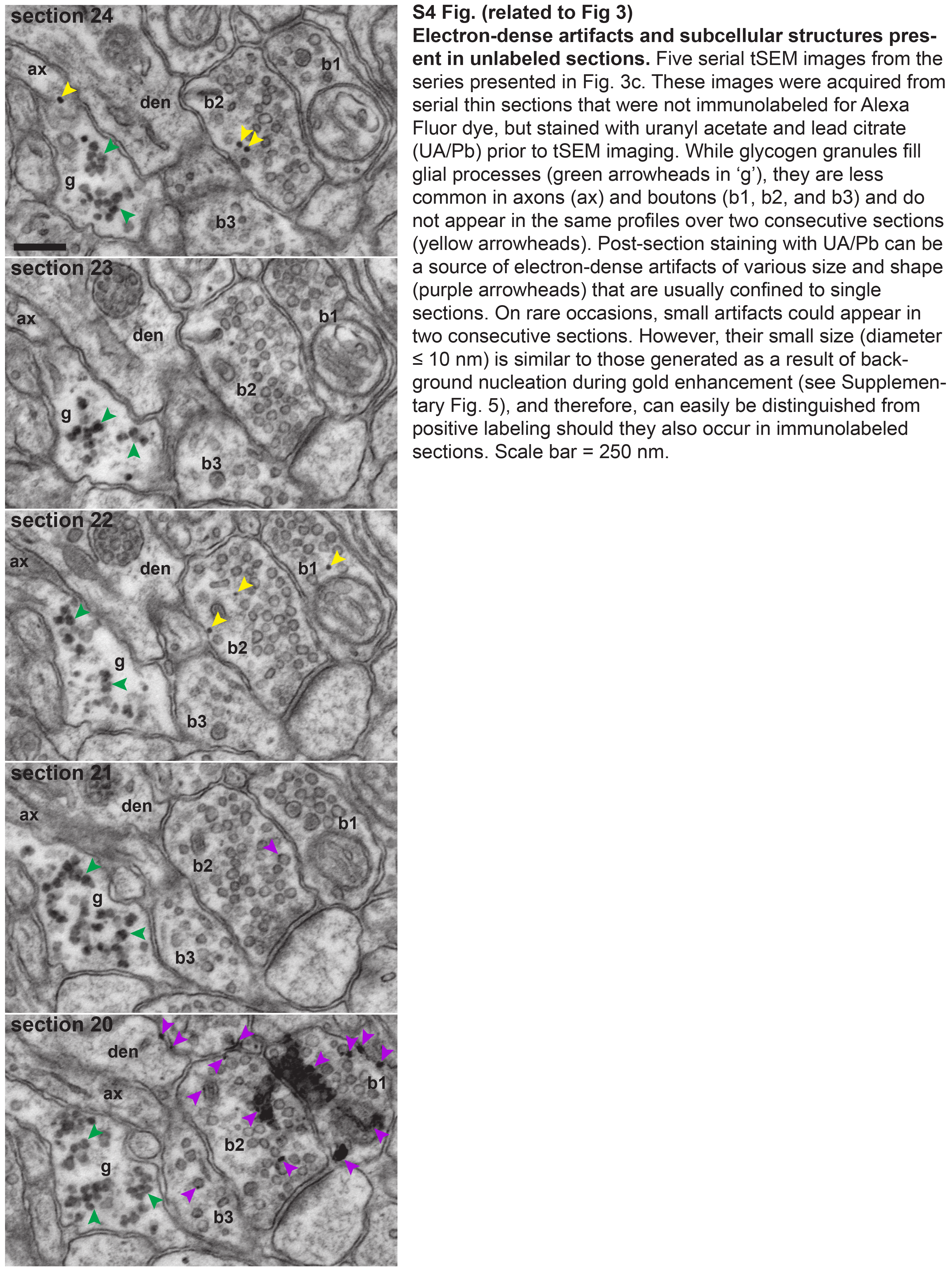

Supplement: S4 Fig — Electron-dense artifacts and subcellular structures present in unlabeled sections. Five serial tSEM images from the series presented in Fig 3C. These images were acquired from serial thin sections that were not immunolabeled for Alexa Fluor dye, but stained with uranyl acetate and lead citrate (UA/Pb) prior to tSEM imaging. While glycogen granules fill glial processes (green arrowheads in ‘g’), they are less common in axons (ax) and boutons (b1, b2, and b3) and do not appear in the same profiles over two consecutive sections (yellow arrowheads). Post-section staining with UA/Pb can be a source of electron-dense artifacts of various size and shape (purple arrowheads) that are usually confined to single sections. On rare occasions, small artifacts could appear in two consecutive sections. However, their small size (diameter ≤ 10 nm) is similar to those generated as a result of background nucleation during gold enhancement (see S5 Fig), and therefore, can easily be distinguished from positive labeling should they also occur in immunolabeled sections. Scale bar = 250 nm. (TIF) [file pone.0226797.s004.tif]

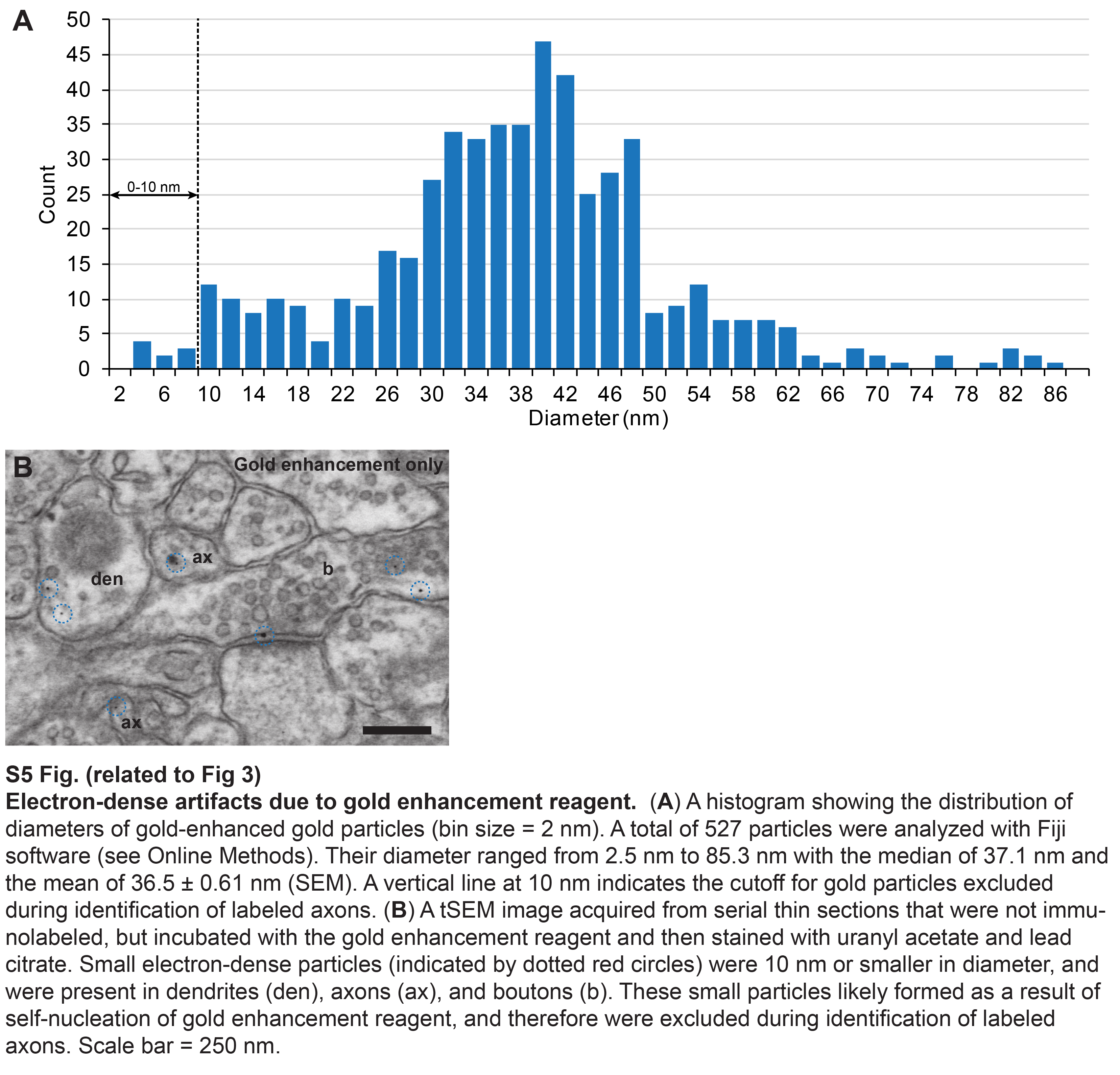

Supplement: S5 Fig — Electron-dense artifacts due to gold enhancement reagent. (A) A histogram showing the distribution of diameters of gold-enhanced gold particles (bin size = 2 nm). A total of 527 particles were analyzed with Fiji software (see Materials and methods). Their diameter ranged from 2.5 nm to 85.3 nm with the median of 37.1 nm and the mean of 36.5 ± 0.61 nm (SEM). A vertical line at 10 nm indicates the cutoff for gold particles excluded during identification of labeled axons. (B) A tSEM image acquired from serial thin sections that were not immunolabeled, but incubated with the gold enhancement reagent and then stained with uranyl acetate and lead citrate. Small electron-dense particles (indicated by dotted red circles) were 10 nm or smaller in diameter, and were present in dendrites (den), axons (ax), and boutons (b). These small particles likely formed as a result of self-nucleation of gold enhancement reagent, and therefore were excluded during identification of labeled axons. Scale bar = 250 nm. (TIF) [file pone.0226797.s005.tif]
